# Racial Disparities in Bowel Preparation and Post-Operative Outcomes in Colorectal Cancer Patients

**DOI:** 10.3390/healthcare12151513

**Published:** 2024-07-30

**Authors:** Alexandra E. Hernandez, Matthew Meece, Kelley Benck, Gianna Bello, Carlos Theodore Huerta, Brianna L. Collie, Jennifer Nguyen, Nivedh Paluvoi

**Affiliations:** 1 Department of Surgery, University of Miami Health System, Miami, FL 33136, USA; matthew.s.meece@gmail.com (M.M.); cth62@med.miami.edu (C.T.H.); blc110@med.miami.edu (B.L.C.); 2 DeWitt Daughtry Family Department of Surgery, Jackson Health System, Miami, FL 33136, USA; 3 Miller School of Medicine, University of Miami, Miami, FL 33136, USA; kelley.benck@med.usc.edu (K.B.); gianna.bello@rutgers.edu (G.B.); 4 Surgical Health Outcomes Consortium (SHOC), AdventHealth Digestive Health Institute, Orlando, FL 32806, USA; nguy0989@gmail.com; 5 Division of Colorectal Surgery, Department of Surgery, University of Miami, Miami, FL 33136, USA

**Keywords:** health disparities, colorectal surgery, post-operative outcomes

## Abstract

Background: Combined pre-operative bowel preparation with oral antibiotics (OAB) and mechanical bowel preparation (MBP) is the current recommendation for elective colorectal surgery. Few have studied racial disparities in bowel preparation and subsequent post-operative complications. Methods: This retrospective cohort study used 2015–2021 ACS-NSQIP-targeted data for elective colectomy for colon cancer. Multivariate regression evaluated predictors of post-operative outcomes: post-operative ileus, anastomotic leak, surgical site infection (SSI), operative time, and hospital length of stay (LOS). Results: 72,886 patients were evaluated with 82.1% White, 11.1% Black, and 6.8% Asian or Asian Pacific Islander (AAPI); 4.2% were Hispanic and 51.4% male. Regression accounting for age, sex, ASA classification, comorbidities, and operative approach showed Black, AAPI, and Hispanic patients were more likely to have had no bowel preparation compared to White patients receiving MBP+OAB. Compared to White patients, Black and AAPI patients had higher odds of prolonged LOS and pro-longed operative time. Black patients had higher odds of post-operative ileus. Conclusions: Racial disparities exist in both bowel preparation administration and post-operative complications despite the method of bowel preparation. This warrants exploration into discriminatory bowel preparation practices and potential differences in the efficacy of bowel preparation in specific populations due to biological or social differences, which may affect outcomes. Our study is limited by its use of a large database that lacks socioeconomic variables and patient data beyond 30 days.

## 1. Introduction

In colorectal surgery, pre-operative bowel preparation has played an important role in mitigating the risks of post-operative complications. Initially, oral antibiotics (OAB) and mechanical bowel preparation (MBP) were widely accepted in the pre-operative period [1,2]. Clinical trials in the early 21st century led to some lack of consensus, with some trials showing MBP failing to protect against post-operative complications [3,4,5], while other studies demonstrated improved surgical outcomes in patients receiving OAB with or without MBP [6,7,8,9]. More recent studies show the use of both MBP and OAB in pre-operative bowel preparation to be associated with reduced incidences of surgical site infection (SSI), post-operative ileus, and anastomotic leaks [10]. Notably, the American Society of Colon and Rectal Surgeons most recent published guidelines that endorse the use of combined OAB and MBP [11]. While many different methods of OAB and MBP are used across clinical practices, a common method includes the administration of multiple doses of oral neomycin and metronidazole (OAB) and the use of polyethylene glycol (MBP) over the course of the day prior to surgery [12]. Historically, MBP is used to clear the bowel lumen of stool and leave only gas, while OAB may reduce bacterial load [13].

In patients with colorectal cancer requiring surgery, surgical complications, such as leaks, are associated with poor outcomes and higher mortality [14,15]. Although current recommendations on bowel preparation are clear, studies continue to demonstrate racial and ethnic disparities in provider instructions and in patient compliance with bowel preparation for other indications, including colonoscopy [16,17]. A study by Stroever et al. found in a national cohort that about 20% of patients did not undergo bowel preparation before elective colectomy, with racial disparities in bowel preparation rates and an overall upward trend in bowel preparation rates over time [18]. However, this has not been studied specifically in a cohort of exclusively colorectal cancer patients, nor have the subsequent post-operative outcomes between different racial and ethnic groups been analyzed [14,15,19]. This is an important issue in colorectal cancer patients specifically, as a recent study found that combined mechanical and oral antibiotic bowel preparation was independently associated with improved recurrence-free survival in colorectal cancer patients undergoing surgery [20]. Additionally, Black patients have the highest rates of mortality in colorectal cancer; the etiology of these differences must be elucidated [19].

To fill this critical knowledge gap, our study aimed to evaluate racial and ethnic disparities amongst bowel preparation administration and subsequent post-operative outcomes in a validated, national cohort of colorectal cancer patients. We hypothesize that minority groups undergoing colorectal cancer surgery receive bowel preparation at a disproportionally lower rate, and that these groups have a subsequent increase in surgical complications.

## 2. Materials and Methods

### 2.1. Study Design

This retrospective cohort study used the 2015–2021 American College of Surgeons National Surgical Quality Improvement Program (ACS-NSQIP)-targeted colectomy database to evaluate post-operative outcomes for patients undergoing elective colectomy for colorectal cancer. The NSQIP provides patient data from more than 400 hospitals. The registry contains retrospective data on each patient, including demographic information; patient outcomes; and pre-operative, intra-operative, and post-operative variables for patients undergoing major surgical procedures in both the inpatient and outpatient settings. The NSQIP database benefits from several unique features of data collection, i.e., the trained/certified collectors, the standardized variable definitions, and the diversity of participating hospitals in the program.

**Figure 1:**
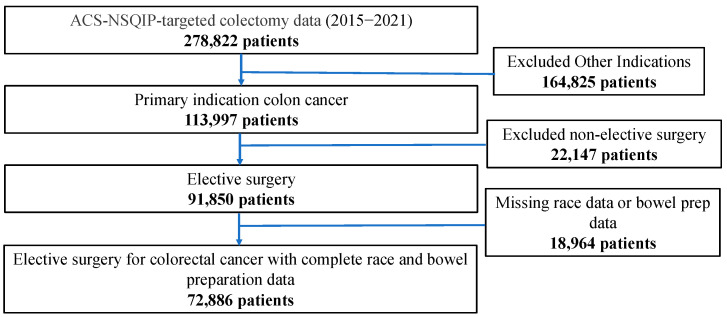
Study Flow Diagram.

All patients undergoing elective colorectal resection for colorectal cancer from 2015 to 2021 were included in our study. Patients who underwent non-elective operation or those without bowel preparation data for OAB or MBP or race data were excluded (Figure 1). This study followed the Strengthening the Reporting of Observational Studies in Epidemiology (STROBE) reporting guidelines [21]. Because the data are publicly available and de-identified, the study was deemed exempt from IRB approval.

Variables collected included age, sex, race, ethnicity, BMI, diabetes status, American Society of Anesthesiologists (ASA) physical status classification, comorbidities (ascites, presence of congestive heart failure 30 days pre-operatively, dependence on dialysis pre-operatively), and planned operative approach (open versus laparoscopic or robotic). ASA status is a descriptive classification to group patients based on their medical comorbidities, ranging from mild systemic disease (ASA2), severe systemic disease (ASA3), severe systemic disease that is a constant threat to life (ASA4), and moribund patients not expected to survive without surgical intervention (ASA5) [22]. Variables were selected based upon prior published research, clinical intuition, and statistical model fit.

### 2.2. Outcomes

Our primary outcome was type of bowel preparation given, categorized as both MBP and OAB, either MBP or OAB, and neither. Our secondary outcomes were post-operative complications of anastomotic leak, post-operative ileus, surgical site infection, prolonged operative time (>3 h versus ≤3 h), and prolonged hospital length of stay (LOS), defined as greater than 30 days. Anastomotic leak was defined as leak of enteral content through an anastomosis determined by the colectomy-targeted database. Post-operative ileus was defined as prolonged nasogastric tube use. Surgical site infection included superficial, deep, and organ space infections. Operative time, given as a continuous measure by the colectomy database, was coded as less than 3 h or greater than or equal to 3 h, based on prior studies [23]. Prolonged LOS is a variable given by the colectomy database. Outcomes were dichotomized as yes or no.

### 2.3. Statistical Analysis

Descriptive statistics were performed for the entire sample. A chi-square test or Fisher’s exact test was used to compare proportions for categorical variables as appropriate. For the continuous variables, normality of the data was assessed using the Shapiro–Wilk test. Depending on the normality results, either parametric (*t*-test) or non-parametric (Mann–Whitney U test) analyses were conducted accordingly. Multiple comparisons were adjusted for using a Bonferonni correction. Multinomial regression was used to assess predictors of type of bowel preparation. Multivariate logistic regression was used to evaluate the impact of race on post-operative ileus, anastomotic leak, SSI, prolonged operative time, and prolonged LOS. A correlation matrix was used to ensure no multicollinearity in the final models. All regression estimates with 95% confidence intervals are reported as fully adjusted results. Statistical significance was set at a 2-tailed *p* value < 0.05. All analyses were conducted using IBM SPSS Statistics (Version 28).

## 3. Results

### 3.1. Patient Characteristics by Race

Of the 72,886 patients evaluated, 82.1% (*n* = 59,805) were White, 11.1% (*n* = 8108) Black, and 6.8% (*n* = 4973) were Asian or Asian Pacific Islander (AAPI) (Table 1). In the total cohort, 4.2% (*n* = 3051) were Hispanic and 51.4% (*n* = 37,463) were male. Amongst racial groups, there was a significant variation in age, with Black patients presenting at a younger age with a mean age of 62 (SD 12) compared to AAPI patients with a mean age of 63 (SD 13), and White patients with a mean age of 65 (SD 13) (*p* < 0.001). Sex distribution also varied significantly, with Black patients having a higher proportion of females (52.2%) in comparison to AAPI (48.0%) and White patients (48.2%) (*p* < 0.001). Black patients had a higher proportion in ASA 4–5 (5.7%), while White patients had a higher percentage in ASA 3 (63.0%) compared to AAPI patients (ASA 4–5: 1.7%, ASA 3: 42.2%) (*p* < 0.001). Lastly, a significant difference in BMI was observed, with AAPI patients demonstrating a lower mean BMI (24.7 (SD 4.9)) in contrast to Black (30.1 (SD 7.6)) and White (29.0 (SD 6.9)) patients (*p* < 0.001). Additional comorbidities can be seen in Table 1.

### 3.2. Bowel Preparation

Significant differences were observed in the distribution of bowel preparation methods. Among those reported using no bowel preparation, Black patients had the highest proportion with 24.9% (*n* = 2016) compared to AAPI (21.9% (*n* = 1089)) and White patients (22.6% (*n* = 13,513)), (*p* < 0.001). AAPI patients were more likely to use one form of bowel preparation, either MBP or OAB, (31.6% (*n* = 1572)) compared to Black (22.0% (*n* = 1783)) and White patients (21.0% (*n* = 12,586)) (*p* < 0.001). Finally, a higher proportion of White patients (56.4% (*n* = 33,706)) used both MBP and OAB compared to Black patients (53.1% (*n* = 4309)) and AAPI patients (46.5% (*n* = 2312)) (*p* < 0.001).

On univariate multinomial regression assessing type of bowel preparation, Black, AAPI, and Hispanic patients were more likely than White patients to have had no bowel preparation compared to combined bowel preparation (MBP+/OAB+) [OR Black 1.17 (1.10–1.24), *p* < 0.001; OR AAPI 1.18 (1.09–1.27), *p* < 0.001; OR Hispanic 1.10 (1.01–1.20), *p* = 0.032]. Black and AAPI patients were also more likely than White patients to have had only one form of bowel preparation (either MBP or OAB) versus combined bowel preparation [OR AAPI 1.82 (1.70–1.95), *p* < 0.001, OR Black 1.11 (1.05–1.18), *p* = 0.005]; however, Hispanic patients were less likely to receive only one compared to combined [OR 0.81 (0.74–0.90), *p* < 0.001].

On multivariate regression, results were similar. Black, AAPI, and Hispanic patients were more likely than White patients to have had no bowel preparation compared to MBP+/OAB+ [OR Black 1.15 (1.09–1.23), *p* = 0.007; OR AAPI 1.16 (1.07–1.26), *p* < 0.001; OR Hispanic 1.16 (1.06–1.28), *p* < 0.001] even after accounting for age, sex, ASA classification, comorbidities, and operative approach (Table 2). Additionally, Black and AAPI patients were more likely than White patients to have had only one form of bowel preparation (either MBP or OAB) compared to MBP+/OAB+ [OR Black 1.10 (1.03–1.17), *p* = 0.005; OR AAPI 1.82 (1.69–1.96), *p* < 0.001] (Table 2).

### 3.3. Post-Operative Complications

**Figure 2:**
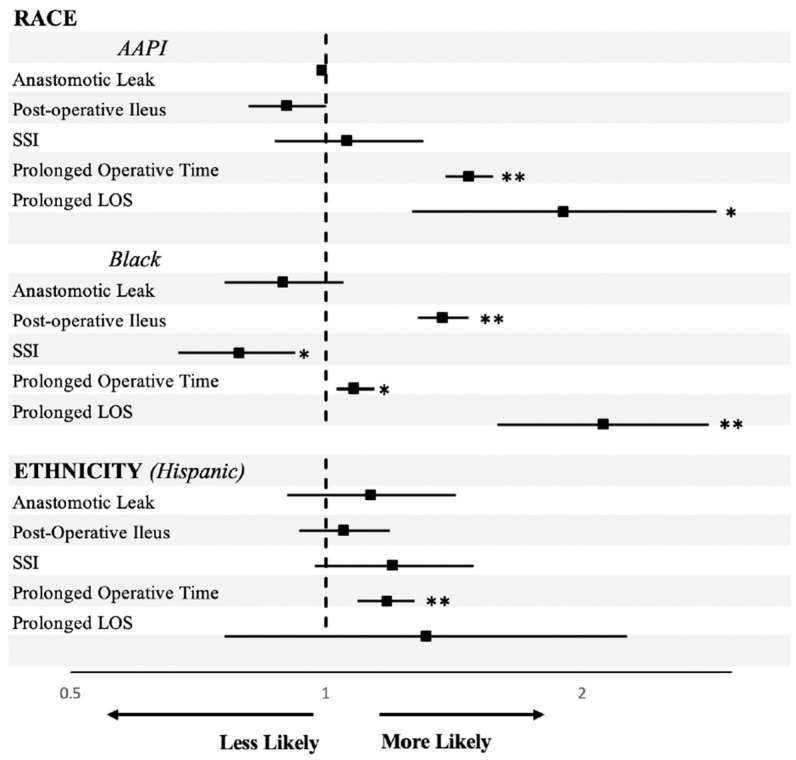
Odds ratios for post-operative complications by race and ethnicity groups compared to non-Hispanic White patients. Odds ratios are adjusted for age, sex, ASA classification, BMI, diabetes, congestive heart failure, ascites, dialysis dependence, bowel preparation received, and planned operative approach. * *p* < 0.05; ** *p* < 0.01.

For our secondary outcomes, using a multivariate regression analysis, we examined the association between race and ethnicity and post-operative complications, adjusting for age, sex, ASA classification, comorbidities, and operative approach. Compared to White patients, Black and AAPI patients had significantly higher odds of experiencing a prolonged hospital stay [OR Black 2.12 (1.59–2.82), *p* < 0.001; OR AAPI 1.90 (1.26–2.88), *p* = 0.002]. For post-operative ileus, Black patients exhibited significantly higher odds, with an OR of 1.37 (1.28–1.47, *p* < 0.001), whereas AAPI patients did not show a significant association (OR 0.90 (0.81–1.00), *p* = 0.042). Additionally, Black and AAPI patients had significantly higher odds of prolonged operative time compared to White patients, with an OR of 1.08 (1.03–1.14, *p* = 0.002) for Black patients and an OR of 1.47 (1.38–1.57, *p* < 0.001) for AAPI patients. Black patients had decreased odds of developing an SSI [OR 0.79 (0.67–0.92), *p* = 0.003]. There were no significant differences in anastomotic leak rates between races or ethnicities (Table 3). Hispanic patients had a significantly higher chance of prolonged operative time compared to White patients [OR Hispanic 1.18 (1.09–1.27), *p* < 0.001], but did not have significant ORs for any other outcomes (anastomotic leak, post-operative ileus, SSI, or prolonged hospital LOS) (Table 3). These results are visually represented in Figure 2.

## 4. Discussion

With a nationally representative sample, we found that racial disparities exist in both rates of bowel preparation administration and certain post-surgical complications, including post-operative ileus, increased operative time, and increased hospital LOS despite the method of bowel preparation. While only one other study by Stroever et al. to date has shown racial disparities in bowel preparation for elective colectomy, we are the first to include both Black and AAPI patients in our analysis, as well as the first to look at the associated post-operative complications [18]. These race-based differences are especially important, as Black patients in the United States (US) have the highest incidence and mortality from colorectal cancer [19]. Moreover, the previous study combined bowel preparation into “any bowel prep received”, while our study goes further to describe the combination bowel prep and single-agent bowel prep groups.

Factors impacting the likelihood of adequate pre-operative bowel preparation are well studied, with some studies showing differences amongst Black and White patients; however, limited focus has been placed on the actual method of bowel preparation given by race [16,24]. Our findings that racial disparities exist in pre-operative bowel preparation are concordant with other literature on bowel preparation given, as well as studies that evaluate the adequacy of bowel prep for elective colectomies [18], prior to colonoscopies [16,24], or pre-operatively for other abdominal surgeries [17]. A similar study by Kane et al. found that amongst a national cohort undergoing elective colectomy, patients who received combined preparation tended to be younger, male, of White race, and of non-Hispanic ethnicity [25]. However, after they controlled for covariates, male gender, BMI over 30, independent functional status, and laparoscopic and robotic surgical approaches were more likely to receive combined bowel prep, while Asian race, hypertension, disseminated cancer, and inflammatory bowel disease were less likely to receive combined preparation. Our study builds upon the current data in its detailed analysis of the specific bowel preparation regimen and in its evaluation of colectomy-related complications for colorectal cancer patients. While other studies demonstrate greater post-operative complications in Black patients [26,27,28], they fail to isolate each complication and only compare Black versus White, as opposed to including other races and ethnicities. Additionally, even though AAPI patients generally have better survival in colorectal cancer than White or Black patients, we still found disparities in bowel preparation administration and post-operative outcomes [29].

While racial disparity in bowel preparation administration is not necessarily surprising given the known racial and ethnic disparities in outcomes and survival in many common cancers, it is important to highlight targetable areas where disparities exist. The underlying reasons are multi-factorial. On an individual level, patient socioeconomic factors that create access to care barriers, such as insurance status, employment, housing, and poverty, likely contribute to the observed differences in our study. A study by Lebwohl et al. found that Medicaid patients had decreased rates of adequate bowel preparation compared to non-Medicaid patients, and within the Medicaid cohort, Hispanic patients had lower adherence; however, this discrepancy was not statistically significant [26]. Interestingly, our study demonstrated higher rates of combined bowel prep vs. single-agent bowel prep in the Hispanic population. Although we controlled for comorbidities that may affect bowel preparation administration, additional individual patient health factors may also contribute, as patients with higher comorbidity profiles have been found to have decreased rates of receiving combined bowel preparation [25]. In addition, individual social differences may act as barriers, such as health literacy, cultural beliefs, language differences [17], patient education [27,28], and patient-provider trust [30]. These differences may also be due to provider practices throughout the nation. A study by McChesney et al. surveyed colorectal surgeons nationally and found that the majority of respondents use pre-operative OAB, and almost all respondents routinely use MBP [31]. These findings did not differ by geographic region or practice setting. Additionally, provider-related factors like implicit bias, cultural competency, and communication style could affect the quality of patient education and shared decision-making regarding bowel preparation [32]. At the systemic level, healthcare policy influences insurance coverage and access to healthcare facilities, which create barriers to receiving adequate pre-operative care, including bowel preparation [33]. Additionally, systemic racism can manifest in healthcare settings through biased treatment protocols, unequal resource allocation, and discriminatory practices, all of which can contribute to racial disparities in surgical outcomes [34].

In addition to the differences in bowel preparation administration rates, our study also found significant differences in post-operative complications and LOS. While racial differences in post-operative outcomes have been described in the literature for broad surgical subspecialties [35] and inflammatory bowel disease populations [36,37], few have described this in colorectal cancer patients receiving surgery [38]. The most striking differences in outcomes were post-operative ileus, operative time, and hospital LOS, which were all increased in Black compared to White patients, even after accounting for use of bowel preparation. Absence of pre-operative bowel preparation is an independent predictor of post-operative ileus [39]. However, in our study, even when controlling for bowel preparation, we found that Black patients had a higher rate of post-operative ileus. Similarly, Wahl et al. reported greater ileus rates in Black patients undergoing elective colorectal surgery [40]. Thus, other aspects of racial disparities may play an independent role in ileus formation or the provider decision to remove nasogastric tubes; however, more research into this association is needed for further conclusions. Our study also found increased operative times amongst Black and AAPI patients. Increased operative times are associated with post-operative morbidity in colectomy patients [41]. While studies have shown longer operative times in Black patients undergoing colectomy for cancer [42], no studies to our knowledge have described this result in AAPI patients. Several studies have also found increased LOS in Black patients compared to White patients; however, only one evaluated the colorectal cancer population, and they did not show a longer post-operative stay for AAPI compared to White patients [38,43]. Interestingly, we found Black patients were less likely to experience SSI than their White and AAPI counterparts, which is similar to findings from a study by Qi et al., finding lower rates of post-colectomy SSI in Black patients [44]. However, other studies have had contradictory findings [45,46]. No studies to our knowledge have reported anastomotic leak rate differences by race in colorectal cancer, with one study on patients with ulcerative colitis undergoing ileal pouch-anal anastomosis showing no differences in leak by race [45].

Differences in post-operative outcomes are influenced by complex, multilevel factors within health systems and hospitals as well as amongst providers and patients. While the data continue to demonstrate disparities in the use of bowel preparation [35,36,37,38] despite its broad acceptance as the gold standard for elective colectomies [11], few studies have examined the subsequent complications of racial and ethnic minorities, as ours has. Initiatives such as following the Enhanced Recovery After Surgery (ERAS) protocols have been shown to decrease racial disparities in some post-operative outcomes [40], and the use of clinical decision-making tools may remove provider implicit bias [47]. Continued research is essential to understanding and confronting racial and ethnic disparities on patient, provider, and health system levels. Our study highlights a directly modifiable target, bowel preparation, that hospitals and providers can work to improve upon.

Our study has limitations, primarily due to the use of the ACS-NSQIP database. The registry only follows patients for 30 days, so complications beyond this period are not captured. Additionally, as with many other large national databases, missingness is an issue that may bias the sample, as this leads to exclusion from the analysis. Notably, the database does not record the use of pre-operative intravenous antibiotic prophylaxis, which while this a standard treatment, its implementation and efficacy in all cases cannot be assumed. Furthermore, there is no standardized bowel preparation protocol in the database, potentially leading to variations in the provided preparation methods. When evaluating racial disparities, it is vital to consider socioeconomics factors such as insurance, income, and other access to care barriers, which are not provided by the ACS-NSQIP data and are important to study in other databases. As the database used lacks socioeconomic variables, some of the racial and ethnic differences in bowel prep and outcomes may be attributed to socioeconomic status, especially given that minority populations tend to have lower SES compared to White patients.

In this study, we chose to examine bowel preparation as an outcome variable to understand the factors that influence its use. While bowel preparation is often studied as an independent variable affecting post-operative complications, understanding the determinants of bowel preparation itself is crucial for addressing disparities in surgical care, as pre-operative preparation is an important and modifiable factor on the cancer care continuum. Pre-operative factors, such as bowel preparation, are not merely independent variables but also important outcomes that reflect the quality and equity of care provided to patients. Our study highlights racial disparities in bowel preparation, suggesting that certain patient groups may not be receiving optimal pre-operative care. This finding has important implications for addressing long-term disparities in surgical outcomes, as inadequate bowel preparation can increase the risk of complications and negatively impact patient recovery as well as survival. By focusing on bowel preparation as an outcome, we aim to shed light on the root causes of these disparities and inform interventions to improve the quality and equity of surgical care for all patients.

## 5. Conclusions

Overall, this large, nationally representative study showed important differences in pre-operative bowel preparation and post-operative complications amongst minority racial and ethnic groups, some of which have not preciously been described. There is a clear lack of research in this area despite the well-documented racial disparities in cancer outcomes. This warrants further exploration into discriminatory bowel preparation practices and variances in the efficacy of bowel preparation in specific populations due to biological or social differences which may affect post-operative outcomes.

## Figures and Tables

**Table 1:** Patient characteristics by self-identified race.

		AAPI	Black	White	*p*-Value
		Column N %	Column N %	Column N %	
Age (mean (SD))		63 (13)	62 (12)	65 (13)	<0.001
Bowel Preparation	None	1089 (21.9%)	2016 (24.9%)	13,513 (22.6%)	<0.001
	Either MBP or OAB	1572 (31.6%)	1783 (22.0%)	12,586 (21.0%)	
	MBP and OAB	2312 (46.5%)	4309 (53.1%)	33,706 (56.4%)	
Sex	Female	2388 (48.0%)	4229 (52.2%)	28,805 (48.2%)	<0.001
	Male	2585 (52.0%)	3878 (47.8%)	31,000 (51.8%)	
Ethnicity	Non-Hispanic	4911 (99.5%)	7855 (99.0%)	56,104 (95.0%)	<0.001
	Hispanic	25 (0.5%)	81 (1.0%)	2945 (5.0%)	
ASA Class	ASA 4–5	85 (1.7%)	461 (5.7%)	2771 (4.6%)	<0.001
	ASA 3	2099 (42.2%)	5107 (63.0%)	35,465 (59.4%)	
	ASA 1–2	2788 (56.1%)	2536 (31.3%)	21,514 (36.0%)	
BMI (mean (SD))		24.7 (4.9)	30.1 (7.6)	29.0 (6.9)	<0.001
Diabetes Mellitus	Yes	1145 (23.0%)	2111 (26.0%)	10,700 (17.9%)	<0.001
	No	3828 (77.0%)	5997 (74.0%)	49,105 (82.1%)	
Ascites	Yes	20 (0.4%)	23 (0.3%)	247 (0.4%)	0.221
	No	4953 (99.6%)	8085 (99.7%)	59,558 (99.6%)	
Congestive Heart Failure	Yes	23 (0.5%)	163 (2.0%)	892 (1.5%)	<0.001
	No	4950 (99.5%)	7945 (98.0%)	58,913 (98.5%)	
Currently on Dialysis	Yes	27 (0.5%)	132 (1.6%)	204 (0.3%)	<0.001
	No	4946 (99.5%)	7976 (98.4%)	59,601 (99.7%)	
Planned Operative Approach	Open	706 (15.7%)	1547 (21.1%)	10,921 (20.0%)	<0.001
	Lap or Robotic	3778 (84.3%)	5793 (78.9%)	43,622 (80.0%)	
Anastomotic Leak	No	4843 (97.7%)	7900 (97.6%)	58,177 (97.4%)	0.311
	Yes	115 (2.3%)	195 (2.4%)	1555 (2.6%)	
Post-Operative Ileus	No	4484 (90.3%)	6883 (85.0%)	52,677 (88.2%)	<0.001
	Yes	481 (9.7%)	1214 (15.0%)	7037 (11.8%)	
Surgical Site Infection	No	4850 (97.5%)	7908 (97.5%)	58,118 (97.2%)	0.08
	Yes	123 (2.5%)	200 (2.5%)	1687 (2.8%)	

**Table 2:** Multinomial regression predicting type of bowel preparation.

Bowel Preparation (Reference OAB and MBP)	No Bowel Prep		Either MBP or OAB	
Factor	OR (95% CI)	Sig.	OR (95% CI)	Sig.
Race				
AAPI	1.16 (1.07–1.26)	<0.001	1.82 (1.69–1.96)	<0.001
Black	1.15 (1.09–1.23)	<0.001	1.10 (1.03–1.17)	0.005
White	Reference		Reference	
Ethnicity				
Hispanic	1.16 (1.06–1.28)	0.001	0.91 (0.82–1.01)	0.07
Non-Hispanic	Reference		Reference	

Note: This model controlled for age, sex, ASA classification, BMI, DM, ascites, CHF, dialysis status, and planned operative approach.

**Table 3:** Multivariate regression for post-operative outcomes.

	Leak		Ileus		SSI		OR Time		Prolonged Hospital Stay	
Factor	OR (95% CI)	Sig.	OR (95% CI)	Sig.	OR (95% CI)	Sig.	OR (95% CI)	Sig.	OR (95% CI)	Sig.
Race										
AAPI	0.91 (0.74–1.12)	0.36	0.90 (0.81–1.00)	0.04	1.06 (0.87–1.30)	0.57	1.47 (1.38–1.57)	<0.001	1.90 (1.26–2.88)	0.002
Black	0.89 (0.76–1.05)	0.16	1.37 (1.28–1.47)	<0.001	0.79 (0.67–0.92)	0.003	1.08 (1.03–1.14)	0.002	2.12 (1.59–2.82)	<0.001
White										
Ethnicity										
Hispanic	1.13 (0.90–1.42)	0.278	1.05 (0.93–1.19)	0.398	1.20 (0.97–1.49)	0.1	1.18 (1.09–1.27)	<0.001	1.31 (0.76–2.26)	0.331
Non-Hispanic										

Note: This model controlled for age, sex, ASA classification, BMI, DM, ascites, CHF, dialysis status, bowel preparation received, and planned operative approach.

## Data Availability

Data were obtained from a publicly available data set.
